# Searching for human connection to transcend symbolisms in pediatric palliative care

**DOI:** 10.1590/0034-7167-2022-0476

**Published:** 2023-06-26

**Authors:** Patrícia Luciana Moreira-Dias, Larissa Fernandes Franco, Maria Aparecida Bonelli, Esther Angélica Luiz Ferreira, Monika Wernet

**Affiliations:** I Universidade Federal de São Carlos. São Carlos, São Paulo, Brazil

**Keywords:** Palliative Care, Professional-Family Relations, Pediatrics, Child Care, Grounded Theory, Cuidados Paliativos, Relaciones Profesional-Familia, Pediatría, Cuidado del Niño, Teoría Fundamentada, Cuidados Paliativos, Relações Profissional-Família, Pediatria, Cuidado da Criança, Teoria Fundamentada

## Abstract

**Objectives:**

to present a theoretical model for the interactional context of health professionals and families of children and adolescents under palliative care.

**Methods:**

qualitative study based on the theoretical frameworks of Grounded Theory and Symbolic Interactionism. Ten palliative care professionals took part in this study through semi-structured interviews employing snowball technique from 2020 to 2021.

**Results:**

the comparative data analysis resulted in the theoretical model “Searching for human connection to transcend symbolisms in pediatric palliative care”. It reveals symbolic elements that substantiate the construction of a collaborative context integrating two phenomena: “Overcoming boundaries and intertwining paths” and “Embracing suffering to weave meaningful experiences”. Symbolisms in palliative care guide the behavior of families and professionals, which makes them the key factor to be managed.

**Final Considerations:**

symbolisms and suffering continually integrate the interactional experience of professionals. Empathy and compassion are fundamental elements to enable their connection with families.

## RESUMO

**Objetivos:**

apresentar um modelo teórico sobre o contexto interacional entre profissionais de saúde e famílias de crianças e adolescentes em cuidados paliativos.

**Métodos:**

estudo qualitativo sob os referenciais da Teoria Fundamentada nos Dados e Interacionismo Simbólico. Participaram dez profissionais atuantes em cuidado paliativo, por meio de entrevista semiestruturada, a partir da técnica “bola de neve”, entre os anos 2020 e 2021.

**Resultados:**

a análise comparativa dos dados resultou no modelo teórico ‘Buscando por conexão humana para transcender simbolismos do cuidado paliativo pediátrico’. Evidencia elementos simbólicos que sustentam a construção do contexto colaborativo integrando dois fenômenos: ‘Rompendo fronteiras e entrelaçando caminhos’ e ‘Acolhendo o sofrimento para tecer experiências de vida significativas’. Simbolismos dos cuidados paliativos orientam comportamentos das famílias e profissionais, constituindo-se no principal interveniente a ser manejado.

**Considerações Finais:**

simbolismos e sofrimento integram continuamente a experiência interacional do profissional. Empatia e compaixão são elementos fundamentais para sua conexão com as famílias.

## RESUMEN

**Objetivos:**

presentar un modelo teórico sobre el contexto interaccional entre los profesionales de la salud y las familias de niños y adolescentes en cuidados paliativos.

**Métodos:**

estudio cualitativo bajo la Teoría Fundamentada en los Datos y el Interaccionismo Simbólico. Participaron diez profesionales activos en cuidados paliativos, a través de entrevistas semiestructuradas, con base en la técnica de “bola de nieve”, entre los años 2020 y 2021.

**Resultados:**

el análisis comparativo de los datos resultó en el modelo teórico ‘Buscando la conexión humana para trascender simbolismos de los cuidados paliativos pediátricos’. Evidencia los elementos simbólicos que apoyan la construcción del contexto colaborativo integrando dos fenómenos: ‘Rompiendo fronteras y entrelazando caminos’ y ‘Acogiendo el sufrimiento para tejer experiencias de vida significativas’. Los simbolismos de los cuidados paliativos guían los comportamientos de las familias y los profesionales, constituyendo el principal factor a ser manejado.

**Consideraciones Finales:**

el simbolismo y el sufrimiento son continuamente parte de la experiencia de interacción del profesional. La empatía y la compasión son elementos fundamentales para su conexión con las familias.

## INTRODUCTION

Palliative care (PC) is defined as holistic and active care to individuals of all ages experiencing health-related suffering due to severe diseases. Its objective is to improve the quality of life of patients, relatives, and caregivers through prevention, early identification, and management of physical symptoms and psychological, spiritual, and social suffering. This care may be offered throughout the disease combined with disease-modifying therapies. Palliative care encompasses and respects the values and beliefs of patients and families^(1)^.

Over 21 million children worldwide are estimated to require a palliative approach every year and more than 8 million require some degree of specialized pediatric palliative care (PPC)^(2)^. In Brazil, a study estimated that 473,503 children required a palliative approach each year and 180,238 children would be benefitted every year by some degree of specialized PPC^(2)^. Moreover, the study suggests a tendency for children to live with an advanced disease for longer compared to adults and, consequently, requiring PC for longer periods^(2)^.

Parents caring for children with a life-limiting disease aim to control the symptoms and the disease, to maintain family balance, and to make decisions that consider their values, encouraged by the desire of being good parents and aware of the vulnerability of their child and disease-related suffering^(3)^. Support from the PPC team is related to a higher understanding of the children’s possibilities and of the parental and family role towards them and other family members^(4)^.

The PPC share the precepts of Family-Centered Care (FCC) to account for the importance of family in people’s lives, the fact that it demands care, and the cruciality of collaborative relations with families for achievements in care and health^(5)^. In this context, communication and relations between the professionals and families are the basis of FCC and PPC, with an emphasis on reciprocity^(6)^, which favors elaboration and helps families to fight the situation^(7)^. Making decisions about the life of a child is a continuous process of communication transcending conversations with professionals and considers individual necessities, putting children at the center of the intervention^(8)^. Children and their families interact with the disease-related conditions, formulating opinions and beliefs which influence how they respond to and experience their condition^(9)^.

Therefore, considering the impact of severe diseases on the life of children and their families, the interactional context is one of the biggest challenges to be overcome by professionals. The evidence^(3,5-6,8)^ reveals that the family has a central role, being integrative and active in palliative care. The children’s experience of disease is part of their families’ history and may impact all the family system and community. It sets the interactional context which is shared with the professionals, established when they meet patient and family, building relational care between families and professionals, who make efforts to guarantee the well-being of the children^(4-6,8)^. Since it is essential to discuss these relations, this study poses the question: “How are the interactions between professionals and families in the practice of pediatric palliative care configured?”

## OBJECTIVES

To present a theoretical model of the context of interaction between health professionals and families of children and adolescents under palliative care.

## METHODS

### Ethical aspects

The study received a favorable opinion from the Research Ethics Committee and all the ethical precepts of the existing resolutions were respected. The Informed Consent Form (ICF) was provided with the online questionnaire (Google Forms) sent to the participants. After reading the text, the participant was presented with the option to agree or decline participation in this study. The participants provided their consent and received a copy of the ICF. In the interviews conducted after February 24, 2021, the recommendations of the Official Letter n. 2 CONEP/SECNS/MS, related to research during the pandemic, were abided by.

### Type of study and theoretical and methodological framework

Exploratory, qualitative study guided by Symbolic Interactionism (SI) articulated to Grounded Theory (GT). Symbolic Interactionism^(10)^ proposes that human behavior is processed and continually transformed during social interaction, with the following premises: the meaning of everything we can interact with is processed in social interaction; these meanings are manipulated and modified based on the interpretive processes; the fact that we act based on those meanings. Thus, the interactionist perspective favors the visibility of meanings and actions emerging in contexts of interaction with the families of children under PC. Grounded Theory is a systematic process of qualitative data collection and analysis whose objective is to create a theory to explain and understand phenomena through a constant movement of approaching and distancing from data, from data collection to analysis, and from analysis to collection^(11)^. The COREQ criteria guided and structured the study^(12)^.

### Methodological procedures

The identification of potential participants started among the members of a research group in a public university. From these indications, the first contacts were performed by the first author to invite professionals and present the study’s objectives, in addition to the form of participation. When they manifested interest in sharing their experience, a meeting through a virtual platform was scheduled to obtain consent and conduct or schedule the interview in an agreed-upon date and time. Grounded Theory is guided by theoretical samples in agreement with the development and density of the data. Data collection was terminated upon theoretical saturation, i.e., when the collection of new data did not lead to new theoretical insights and/or did not reveal new properties of the central theoretical categories. The researcher conducted the theoretical sampling by using the sample to develop properties of categories until no new properties could be extracted^(11)^.

### Local

The participants were selected through snowball sampling^(13)^ based on an indication from key informants, which, in this case, started in the study group and, subsequently, based on indications from the participants themselves.

### Data source

The participants of this study were 10 health professionals with experience in providing care to children and adolescents under PC and their families. Seven of these professionals contributed to theory formulation and 3 professionals contributed to the validation of the theoretical model. The inclusion criteria for this study were being a high-level health professional, with experience providing care to children and adolescents under palliative care and their families, with or without an academic specialization in this field. Professionals who presented any condition precluding sharing their experience through a remote interview were excluded.

Regarding professional training, 4 were physicians, 3 were nurses, 2 were psychologists, and 1 was a physiotherapist. All had graduated over 8 years ago, 6 had an academic specialization in PC, 8 of them worked in hospital and outpatient units, 1 in a pediatric intensive care unit, and 1 only in an outpatient unit. The mean age of the interviewees was 41 years old. All but one participant were female.

The first sample group consisted of two professionals (physician and physiotherapist) whose data enabled forming initial categories, out of which hypotheses emerged on the influence of the environment over interactions, symbolisms in palliative care, and the need for a bond with the child. Through an analysis of the first group, more details of the context of interaction emerged and a second sample group was determined. This group comprised three professionals (nurse, physician, and psychologist) and their data rendered the initial categories denser and established new ones, such as the importance of honest communication, the challenges of interacting with other teams providing care to the children, the efforts of professionals to create common objectives through empathy and compassion with families. Through a constant analysis of data concomitant to data collection, the third sample group was formed by two professionals (nurse and physician), with the intention of listing aspects related to the symbolisms of palliative care and the elements sustaining interactions, which enable rendering the categories denser and achieving theoretical saturation. In addition, three professionals (physician, nurse, and psychologist) validated the results and the theory, forming the fourth sample group. In that moment, the theoretical model was presented to the participants individually. Each of them analyzed the representativeness of the analytical categories in relation to their interactional experiences and the resonance of the meanings of this experience. Following a conversation with the researcher on this theme, with no further additions to the model, a verb in the definition of one category was altered, as was the position and colors of one of the elements of the diagram.

The participants were identified with the letter P (for professionals), followed by Arabic numbers indicating the order in which they joined the study.

### Data collection and treatment

In face of COVID-19, the interviews were conducted remotely and individually through meeting platforms (*Zoom* or *Google Meet*), with only their audio recorded, preserving the image of the participants. The interviews, which were semi-structured, were conducted by the first author of this article, who had prior experience with qualitative interviews. The data were collected between 2020 and 2021, totaling 8 hours and 21 minutes in interviews, with a mean duration of 50 minutes each. The interviews started, in all sample groups, with the following instruction: ‘tell me how the families are inserted in your practice of pediatric palliative care’. From this question, new ones were asked for increased data depth and density.

### Data analysis

The data analysis was conducted in parallel to the data collection through coding proposed by Strauss and Corbin^(11)^, comprising three phases: open, axial, and selective. Open coding, the first step for the elaboration of analytic interpretations, involved the specification of each word, line, or data segment to use the most significant or frequent codes to classify, synthesize, integrate, and organize large amounts of data. The first data unfolded into analytic ideas to proceed to a new collection and data analysis. The coded incidents were compared to one another for conceptual similarities and differences and grouped into categories, which expressed the meaning of the codes. The categories were named according to their properties and dimensions. Subsequently, axial coding was structured under the reflection of the Paradigm Model in an analytic process on the level of theory and category, leading to five components: causes, intervening conditions, context, strategies, and consequences; a phase in which the categories were integrated from the comparison between incident and the properties of the category in face of the same phenomenon, achieving theoretical saturation, which is characterized by data repetition and absence of new data. Selective coding, the last analytic movement, was a reduction of the theory, when the researcher formulated the theory through the uniformities of the original set of categories and their properties. Memoranda, researcher registers of ideas, questions, and hypotheses on the possible relations among the data, were formulated and adopted in this study^(11)^.

## RESULTS

The theoretical model *‘Searching for human connection to transcend symbolisms in pediatric palliative care’* is demonstrated with two phenomena: ‘Overcoming boundaries and intertwining paths’ and ‘Embracing suffering to weave meaningful experiences’. Professionals believe and count on the centrality of the families in PPC and their understanding leads to efforts and compromise in thinking and including the family throughout their interactions. ‘Believing in the construction of a context of interaction with the family’ is the causal condition of the model, in which the professional inaugurates and sustains an invitation for families to join the process, which families gradually accept. Joint construction is sustained by sharing between professionals and families, representing the events which lead to the phenomenon ‘Overcoming boundaries and intertwining paths’.

The symbolisms of PC, commonly understood as an approach exclusively targeted at people at the end of their lives and in relation to death, may manifest through the family’s resistance to this type of care. As a social object, these symbolisms are a hindrance to the interaction between professionals and families and are gradually deconstructed with an adequate management of symptoms and suffering, of honest and empathetic communication, of the legitimation of the families’ values and a focus on the children’s quality of life. This movement demands continuous efforts from the professional to be in touch with the family through human connection, a process in which the self is continuously defined and redefined throughout interactions. Professionals use their minds to direct their actions, manipulating their own symbols, talking to themselves and to their selves, being also transformed by an interactional context which is shared and built with the children and their families. Thus, ‘facing a challenging context to palliative care’ shows the conditions in which professionals provide care. To overcome these challenges, professionals gradually ‘creating a basis of interaction with the families’, which represents the professionals’ strategies for action and interaction.

The interaction among symbols, self, and mind enables professionals to put themselves in another’s position, in this case, those of children and their families, and, moved mainly by compassion, be in a constant search for connection with children and their families to build a shared goal for care, involving decision-making and the elaboration of plans and goals. The symbolic interpretation of human action as a social object enables the use of symbolisms and the professionals’ selves in the construction of an interactional context with the families. ‘Mobilizing in face of losses shared with the family’ reveals professionals sharing the perception of the families of their children’s changes, their loss of abilities, and reduced functionality, regarding several alterations of the family dynamics and their previous life, or the life they had planned before the diagnosis. This is the intervening condition which is supported by the interactions with families and the professionals’ experiences related to the phenomenon ‘Embracing suffering to weave meaningful experiences’. The consequence of this interactional process is ‘feeling shared care with the family reverberating within oneself’.

**Figure 1:**
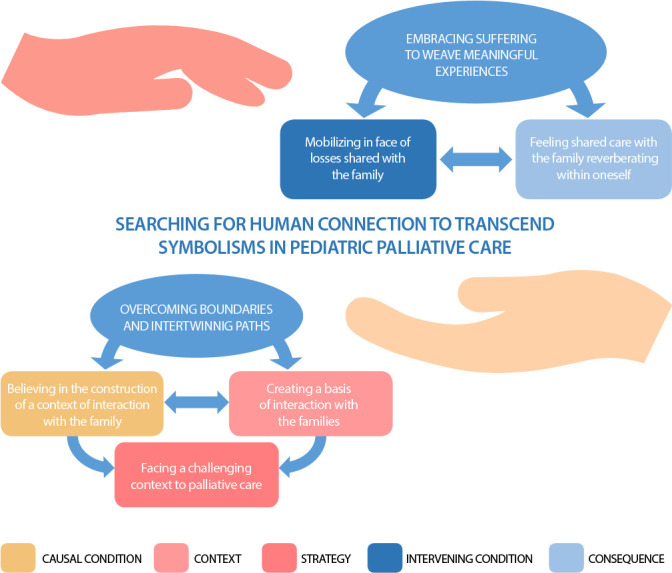
Theoretical model ‘*Searching for human connection to transcend symbolisms in pediatric palliative care’* with its phenomena and categories, São Carlos, São Paulo, Brazil, 2021

The social interaction between professionals and families is a symbolic social action based on the capacity of putting oneself in another’s position and is influenced by the actions of professionals ‘Searching for human connection to transcend symbolisms in pediatric palliative care’, which is the central category of this theoretical model, presented in Figure 1.

**Figure 2:**
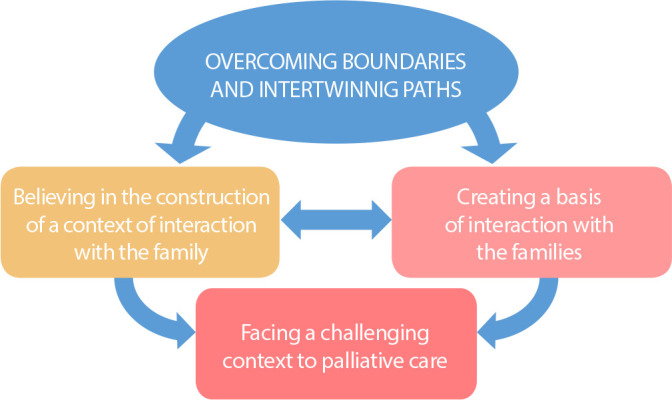
Phenomenon ‘Overcoming boundaries and intertwining paths’, São Carlos, São Paulo, Brazil, 2021

The phenomenon ‘Overcoming boundaries and intertwining paths’, which translates the efforts and processes to achieve a relational context to intertwine and connect with the children’s families, is structured into three categories, as shown in Figure 2.

The category ‘Believing in the construction of a context of interaction with the family’, structured into four subcategories, deals with the actions of the professional to invite and involve the family for a joint construction of care. The conceptual basis ‘Understanding the relevance of collaborative care with the family’ is continuously reported in conversations within and through the self on the necessity of a joint participation of families in PC. Thus, to understand the singularities of the meanings of each family, professionals make themselves available to ‘dive into the singularity of each family’. In this process, they emphasize the relevance of empathy, sensitive listening, authentic presence and availability, and comprehensive efforts in their relations with the families. An observation of their behavior and a search for the families’ voices to reveal their structure, patterns, and communication are conducted as relations take place and gradually reveal the mistaken meaning of PC. Thus, this is aimed at ‘demystifying the symbolisms of palliative care’ of the family. The meaning of PC intervenes in the context’s features related to collaborative relations, since it is articulated to death and leads the family to questions. By embracing these symbolisms and searching for changes in the meaning of PC, the professionals draw closer to the family, leading to an opening for a relationship. To achieve this, they report ‘believing that interaction takes place when one is present and available’, an aspect which favors a welcoming approach, promoting safety and reliability:

*But the surprise when we say “palliative” is due to thinking that they are dying. This is the first surprise people have. It’s about deconstructing this image they bring and building a different one. One in which life doesn’t end here. We are talking about life all the time and planning for it to be as plain as possible.* [...] *It’s a concept, I guess the relationship starts - the first step - with this deconstruction we do.* (P6)

The category ‘Facing a challenging context to palliative care’ includes obstacles to palliative care and the feeling of powerlessness of professionals in face of them. It includes the fact that it is ‘hard to enter the universe of the family’, departing from the professionals’ formative gaps to approach the families, as is referring the children and their families to PC in more advanced stages of the disease. In parallel, they are ‘dealing with obstacles in the relation with other professionals’, a dissonance in the focus of a care restricted to curing the disease. Thus, there are difficulties to providing PC concomitantly to the disease-modifying treatment. The relation of the PC professional with the family becomes fragile and limited, confused, in face of the proposals and approaches. Thus, a ‘context configuring symbolisms and family participation’ is faced with, reducing time and opportunities for offering PC:

*The pediatric ICU teams I worked with mostly want to provide palliative care only in terminal cases. This is one of the things I used to call attention to, one of the issues I called attention to the most in the ICU, in the meetings with the interdisciplinary team* [...] *because sometimes I could see that the patient was eligible: “but he’s not dying”.* [...] *They would say: “fortunately so, because if we talk to the family only when he’s dying we’ll continue to have this culture of ‘everyone under palliative care is here just to die’”.* (P5)

Faced with this scenario, the professionals try to ‘Creating a basis of interaction with the families’, a category which translates the professionals’ choice for an empathetic and compassionate behavior in their effort to clarify the needs of children and their families, always in collaboration with them. ‘Recognizing the centrality of empathy and compassion’ sustains the professionals’ movement to understand the situation, making efforts to comprehend the families’ perspectives. Intense mental work and interactional processes take place within the self with the revelation of the centrality of the children’s well-being to the family. In face of this, a process is started to ‘search for connection with the child to draw closer to the family’ and hence to identify and reveal needs of the children which were invisible to the family. The children’s well-being is a factor which integrates families and professionals and starts, even if as orientation from the professional to the family, through conversations on the possibilities of relieving the children’s suffering. It provides more space to increasingly ‘give visibility by providing palliative care through the management of adverse factors’. The clarification on PC suiting the situation of each child and family favors a more open relation between the professional and the family. However, due to a diversity concerning PC, it is taken as a premise that ‘it is necessary to share responsibilities’. The professional considers all elements of the context and respects the dynamics of its processes, particularly the relational choices of the family and the peers’ pain when dealing with situations of extreme suffering, such as the end of the life of children:

*The interaction with me was not nice, perhaps they could talk to a different professional. So, in that moment, what did we use as a strategy? We would call a different professional the family had contact with or had had contact during their years or months of treatment, to talk.* [...] *Or, for instance, these were families that demanded nothing from the psychologist: “this family is suffering, and they can’t bring it to us, we did not have a good interaction”, she could bring many things to me. But I’m not a psychologist, I can’t address these questions with them. So how were we, as a palliative care multiprofessional team, to meet this demand?* (P3)

**Figure 3:**
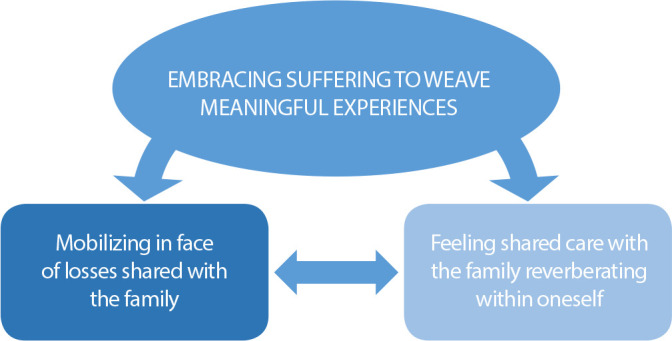
Phenomenon ‘Embracing suffering to weave meaningful experiences’, São Carlos, São Paulo, Brazil, 2021

The phenomenon ‘Embracing suffering to weave meaningful experiences’ represents the interactions of the professional with the family and the self, defined and redefined throughout the process. From the action of the mind in interaction with the self, the professionals weave meanings for a shared experience. These contemplate the categories represented in Figure 3.

The category ‘Mobilizing in face of losses shared with the family’ is composed of three subcategories. The context of interaction shared with the family is permeated by challenging situations in which the professional, mobilizing in face of the family’s suffering, tries to welcome this suffering, ‘providing support by sharing moments of crisis with the families’. It represents a set of attitudes and interventions in moments of major suffering, distress, and uncertainty, as when faced by the child’s worsening conditions, when there are no more proposals of disease-modifying interventions or support in the end of the child’s life, the most painful moment of the family’s experience. In face of these moments of crises, the professional interacts by ‘witnessing the family’s grief in face of their losses’. In this context of interaction, hope emerges as central symbolic resource in this process, in which, supported by the principles of PC, the professional tries to lead the family to dislocate from a passive role to a more active one, ‘legitimizing hope as a family resource’:

*They* [parents] *trust us, because, as rehospitalization is frequent, the children generally have already had many other complications* [...] *but with each hospitalization there is an enormous fear.* [...] *“Is the child leaving the ICU this time?”. And we have to deal with this constant fear families have.* (P1)

The families of the children under PC are also impacted by the interactions established with them, which are configured in their experience as a continuous and dynamic construction in which the professional tries to be in connection with the family. Thus, the consequence of interactive experiences is shown in the category ‘Feeling shared care with the family reverberating within oneself’, composed of two subcategories.

This essentially human connection puts the professional in the position of a suffering human being, sharing grief and hope. To deal with suffering and be able to use it to weave meaningful experiences with the family, the professionals must understand their role, ‘establishing limits for their place in interaction’. Looking into oneself represents a conversation with the self, defining and redefining meanings from interactions built with the family. This process is intensely experienced by professionals providing palliative care to children, ‘transforming through an experience shared with another’:

*Palliative care has shown to me that the more you know yourself, the more you find out about yourself and understand your feelings, your needs, your shortcomings, the more you can separate what is other people’s from what is yours. So, throughout this process, many times I couldn’t deal with it, it was too difficult to deal with other people’s suffering. Because my own suffering would hit me. And then it was all combined.* [...] *And it wouldn’t be possible if I didn’t have also to work on me, right? From therapy, understanding, from understanding my own family, all the times I was there and I didn’t feel ok.* [...] *Including the roles we play in our lives.* (P4)

The central category *‘Searching for human connection to transcend symbolisms in pediatric palliative care’* emerges from the process of interaction between the professional and the family in the context of pediatric palliative care. As a process, it represents the construction of a collaborative interaction sustained by human connection, in which several obstacles are found, mobilized in particular by socially constructed symbolisms about palliative care. Human connection is the central element that sustains interactions even in the most challenging moments, achieved through mutual effort and opening for this relation, in which the search for what is best for the child is the mobilizing agent of the action of the mind.

## DISCUSSION

The results enable showing aspects of the process of interaction between professionals and families in the context of PPC, particularly those related to the proposal and achievement of opening and trust for a collaborative interaction. The symbolisms of PC were shown to be an important intervening factor and, to oppose them, the professional attempts to clarify the concept and approach the family. Connection, empathy, and compassion invite professionals to put themselves in the place of another and see the world from another’s perspective, searching thus to understand - in the singularity of each family and their history - their desires, values, and their sacred, which reinforce the children’s well-being and their quality of life as a common goal between professional and family. This evidence corroborates evidence from other studies^(14-20)^ on the experiences of professionals and families with children under PC.

When patients are children who experience complex medical conditions, partnership is an approach that supports a proactive planning of care, one in which the needs of child and family are met^(14)^. Family participation is a structuring axis of PC: the family is a protagonist, presenting a collaborative attitude that is essential in care^(15)^. Taking care of families with children in life-threatening conditions and, therefore, eligible for PC, is an ethical duty, guided by the idea that children are part of their family’s context. Moreover, families have the right to provide care in face of the suffering and vulnerability of their children, and it is fundamental that professionals take up the duty of listening to the families with compassion, improving communication and enabling parent empowerment and shared decision-making^(16-19)^. Such evidence is close to the presuppositions of Family-Centered Care (FCC), a philosophy of care which is closely related to the principles of PPC. Although they have not been mentioned as such by the participants, the elements of respect to dignity, collaboration, and partnership were present and emerged from the evidence of this study, and believing in their importance for care was shown to be a foundation for professionals through their investment on the construction of a joint project with the family to provide childcare. Certain authors explain that parents value professionals perceived as available, responsible, open for questioning, and focused on care and relief of the children’s suffering. They frequently want to be treated as partners of the team in providing care^(20)^. In this study, the professionals revealed their belief that interaction with the family is possible through presence and availability.

Family-Centered Care is recommended for PC, with the consensus that the unit of care in situations of life-threatening diseases is composed of patient and family, which suggests asking the questions: “Who is the family?”; “When to intervene jointly with the family?”; “Which model should be applied and in which circumstances?”^(21)^. These questions address the professionals’ effort to understand the singularity of each family, as shown in this study, in which the professionals tried to connect with the children, advocating for them and meeting their needs. By legitimizing and giving visibility to these needs, respecting the child as a being under development and using sensitive and compassionate language and approach, the connection with the family is strengthened. The approach of FCC needs to be directed to an approach covering the children’s rights of participating in all aspects of care in accordance with the needs of their families. This perspective is shared in a study in which the family was understood as an emotional unit, a system of interconnected and interdependent individuals. Children cannot be understood in isolation from this system and it is necessary to join efforts to promote fundamental principles and the protection, promotion, and rights to participation, strengthening the perspective of seeing children as representative agents of their own experiences and desire for respect^(22)^. In addition, as an emotional unit, the impact of the experience on the family system, in agreement with the PC principles, must be considered. The evidence reveals psychic suffering and high levels of stress and distress in parents and siblings, in addition to the families’ financial difficulties^(23-24)^.

By inviting the families to collaborative care, believing in the importance of this partnership, the evidence of this study shows that one of the greatest obstacles to be faced by the professional is dealing with the symbolisms of palliative care. The meanings attributed to palliative care, which are built in social interactions, may pose an important obstacle to joint constructions by professionals and families, as well as among the other professionals of the health team. A study has shown that a misunderstanding of the palliative approach was an important barrier and postponed the onset of the children’s care and that feelings of insecurity, fear, resistance, and guilt were present. These feelings were present both to families and professionals, who referred a lack of preparation for understanding and fighting death. In addition, the conceptions of PC involved cultural and religious values of life and death^(25)^.

International studies show that the term “palliative” may provoke an association with end-of-life care, which makes many institutions use alternative terms, such as “supportive care”, to avoid this connotation. Professionals consider that this is important when presenting a team to a family^(26)^. This evidence is shared in this study, in which professionals reveal similar experiences regarding the association with end-of-life or death and with the strategy of introducing themselves without using the term “palliative care”. Additionally, the reality revealed by the professionals is that children are referred to them when there are no more proposals for disease-modifying treatment or when the children are progressing to end-of-life. Studies show that, although professionals recognize the value of the principles of PPC and the benefits of incorporating this approach during the disease’s development, they worry about a possible confusion of roles among the multidisciplinary team and between the family and the team’s professionals^(27-28)^.

### Study limitations

This manuscript is limited by the number of participants, when appreciated in isolation, which may be conceived as a shortcoming; however, the theoretical and methodological framework, the experience of the researchers in studies under these frameworks, and the validation of the theoretical model strengthen the findings and make them relevant evidence for the context of PPC. Moreover, the data collection was performed remotely due to the context of pandemic, an unusual strategy in qualitative interviews.

### Contributions to the field

The evidence presented by this study represents the interactional experience built by professionals with families and children under PC, resulting from the social interaction through which the professional’s self is continuously defined and redefined, transcending socially constructed symbolisms on palliation. In addition, the study enables a view of the unveiled elements, which corroborate and widen current knowledge, with human connections as a symbolic element which emerges from experience and integrates shared interactions at all moments. This study provides fundaments for the essentiality of the philosophy of FCC and the approach of PC for health practices while showing how their lack of use hinders achievements related to well-being and quality of life of children and families. It also gives visibility to the urgency of meeting another to enable care, when empathy, compassion, and comprehensive listening are pointed out as means to partnering up with families and investing, in the social sphere, towards transformations of the meaning of palliation.

## FINAL CONSIDERATIONS

The theoretical model enables understanding the meanings of palliative care which emerge in the interaction of professionals and families, searching for a human connection through a symbolic dialogue of definition and redefinition of the self and through the mental process which enables its action. The interactions among professionals and families are configured in the practice of PPC from an invitation to the process of interaction, in which empathy and compassion are central elements. Professionals create and sustain this invitation for the construction of a collaborative process, investing in human connection. Transcendence is only possible through connection based on respect and singularity, empathetic listening, and compassionate and sensible communication. Believing in the importance of connection with children and their families and the essentiality of a collaborative project shared with the family is a basis for the practice of PPC, the force which sustains the interaction and joins paths in face of the adversities emerging throughout the children’s disease and directed at shared objectives.
